# Hidden Decomposers: the Role of Bacteria and Fungi in Recently Intermittent Alpine Streams Heterotrophic Pathways

**DOI:** 10.1007/s00248-023-02169-y

**Published:** 2023-01-17

**Authors:** L. Gruppuso, J. P. Receveur, S. Fenoglio, F. Bona, M. E. Benbow

**Affiliations:** 1 Department of Life Sciences and Systems Biology, University of Turin, Via Accademia Albertina 13, 10123 Turin, Italy; 2 Centro per lo Studio dei Fiumi Alpini (ALPSTREAM – Alpine Stream Research Center), Ostana, (CN) Italy; 3 Institute for Genome Sciences, University of Maryland, Baltimore, MD USA; 4 Department of Entomology, Michigan State University, East Lansing, MI USA; 5 Department of Osteopathic Medical Specialties, Michigan State University, East Lansing, MI USA; 6 Ecology, Evolution and Behavior Program, Michigan State University, East Lansing, MI USA

**Keywords:** Bacteria, Fungi, Microbiomes, Microbial communities, Leaf litter decomposition, Intermittent rivers, In-stream conditioning, Alpine streams, Necrobiome

## Abstract

The frequency of flow intermittency and drying events in Alpine rivers is expected to increase due to climate change. These events can have significant consequences for stream ecological communities, though the effects of reduced flow conditions on microbial communities of decomposing allochthonous leaf material require additional research. In this study, we investigated the bacterial and fungal communities associated with the decomposition of two common species of leaf litter, chestnut (*Castanea sativa*), and oak (*Quercus robur*). A sampling of experimentally placed leaf bags occurred over six collection dates (up to 126 days after placement) at seven stream sites in the Western Italian Alps with historically different flow conditions. Leaf-associated bacterial and fungal communities were identified using amplicon-based, high-throughput sequencing. Chestnut and oak leaf material harbored distinct bacterial and fungal communities, with a number of taxonomic groups differing in abundance, though bacterial community structure converged later in decomposition. Historical flow conditions (intermittent vs perennial rivers) and observed conditions (normal flow, low flow, ongoing drying event) had weaker effects on bacterial and fungal communities compared to leaf type and collection date (i.e., length of decomposition). Our findings highlight the importance of leaf characteristics (e.g., C:N ratios, recalcitrance) to the in-stream conditioning of leaf litter and a need for additional investigations of drying events in Alpine streams. This study provides new information on the microbial role in leaf litter decomposition with expected flow changes associated with a global change scenario.

**Supplementary Information:**

The online version contains supplementary material available at 10.1007/s00248-023-02169-y.

## Introduction

In low-order streams, allochthonous leaf litter is a fundamental energy source, and its decomposition plays a key role in nutrient and energy cycling [1]; therefore, coarse particulate organic matter (CPOM) decomposition has been widely used to assess river function [2, 3] and is the basis for the River Continuum Concept (i.e., RCC) [4]. However, for intermittent rivers and streams (i.e., IRES), the general assumptions of the RCC do not necessarily apply; in those lotic ecosystems, the flow continuity along the longitudinal dimension is lost, creating a patchy distribution and changes in water physicochemistry [5]. For instance, when dry phases occur, the main agents of organic matter degradation shift from leaching and use by aquatic microorganisms and invertebrates to photodegradation and terrestrial organisms [6, 7].

In general, submerged leaf litter decomposition consists of leaching and mineralization through microbial activity, which then facilitates macroinvertebrate consumption [8, 9] and fragmentation [10], driven by interacting intrinsic (i.e., C:N ratio, leaf texture, and secondary compound content) and extrinsic factors [11, 12]. For extrinsic factors, recent studies [13, 14] reported that 80% of fungi found on decomposing leaves come from the leaf surface (such as *Cladosporium* and *Alternaria* [15]). Some bacteria genera, such as *Pseudomonas*, *Sphingomonas*, and *Massilia* [15], can be detected on the surface of newly senesced leaves and are able to persist under variable environmental conditions; other bacteria colonize leaf litter mostly from the sediment or water column after the initial fungal breakdown of complex molecules [16, 17]. These molecules can be cellulose, hemicellulose, and lignin [18, 19], and their changes influence later-arriving fungal species and overall leaf litter decomposition rates [20–22]. Normally, the decomposition process occurs over the course of weeks in temperate regions of the world [23, 24], while in alpine regions, it can be accelerated by strong current velocity (physical fragmentation enhanced), or delayed due to lower water temperatures and glacial influence assessed through the glaciality index (i.e., developed for European streams that reflects water temperature, conductivity, suspended solids, and channel stability) [25].

Studies have suggested that bacteria and fungi can have both positive and negative interactions during CPOM decomposition [26]. These interactions affect the taxa and relative abundances of leaf litter microbial communities, and thus, the types of nutrients and compounds produced during the decomposition process [27], a phenomenon which can also be altered by flow intermittency. Even before high throughput sequencing tools, early work provided strong evidence of the important functional roles of leaf litter microbial communities [28–30]. Ongoing advances in genomic sequencing technologies now allow for new insights into taxa identification and roles of microbial communities associated with leaf decomposition, including how they vary among leaf sources (e.g., originating taxa [31]), along abiotic gradients (e.g., temperature, water quality, land use effects) and in the presence of other macroconsumers [32, 33]. Furthermore, the increasingly frequent occurrence of low-flow phases and dry events in a wide variety of streams and rivers worldwide has emphasized the need to understand how in-stream microbial communities respond to changing flow conditions. Desiccation due to the absence of flowing water can affect stream microbial communities associated with leaf decomposition, by delaying or severely retarding it, thus disrupting heterotrophic energy pathways [34, 35].

In this study, we investigated aquatic leaf litter microbial communities associated with decomposing leaves of two tree species with different nutrient qualities: high quality and lower recalcitrant chestnut (*Castanea sativa*) and low quality and more recalcitrant oak (*Quercus robur*). Leaf litter bags were used to characterize the decomposition-associated microbial communities at seven sites in three Alpine streams of Italy: the Po River, Pellice River, and Varaita River. These rivers are impacted by recent flow intermittence due to anthropogenic pressures and climate change [36–38]. Leaf litter microbial communities were characterized from leaf disks followed by high throughput sequencing of the 16S rRNA and ITS amplicons for bacteria and fungi, respectively. Our goal was to characterize the variability of microconsumer communities from different leaf litter sources (i.e., tree species) among these three rivers affected by flow intermittency. We hypothesized that: (i) bacterial and fungal communities would be primarily represented by genera typical of the leaf phyllosphere, such as *Pseudomonas*, *Sphingomonas*, *Massilia*, *Cladosporium*, and *Alternaria*, especially early in decomposition; (ii) as leaf litter decomposition progressed, we expected a shift in microbial families from leaf specific taxa, such as Oxalobacteraceae and Pleosporaceae, to litter breakdown specific taxa, such as Hyaloscyphaceae and Helotiaceae; and (iii) leaf litter microbial community composition would be significantly different across river sites that underwent drying events compared to those where the flow was not interrupted.

## Materials and Methods

The field experiment was conducted from 13 December 2018 to 19 April 2019, with sampling occurring every 21 days, which was determined to be an appropriate regime based on our previous Alpine stream work [39, 40]. This provided a total of six sampling dates in three mountain rivers of the Western Italian Alps: the Po River, Pellice River, and Varaita River. There were four sites in the Po river (Crissolo, Ostana, Sanfront, and Revello, upstream-to-downstream respectively, hereafter CRI, OST, SAN, and REV), two in the Pellice River (Pellice M, i.e., upstream and Pellice V, downstream, hereafter PEM and PEV), and one in the Varaita River (hereafter VAR). Sites were selected based on previous experience of research during prolonged drying events (for additional details see [36–38]) and historical discharge data of the Environmental Regional Agency [41]. This approach was chosen because those three Alpine streams (Po, Pellice, and Varaita) have been widely investigated by our research group within the framework of the PRIN NO ACQUA project (Risposte di comuNità e processi ecO-sistemici in corsi d’ACQUA soggetti a intermittenza idrologica). The PRIN NO ACQUA project focused on the effects of hydrological intermittency on biodiversity, functionality, and resilience mechanisms in previously perennial Alpine streams.

The selected sites do not always go dry during summer; they were naturally perennial stretches selected for their changing hydrology and increased intermittency over the last several years, related to decreased precipitation with continued water abstraction. Further information about drying events in the three intermittent sites throughout the experiment is reported in Table 1. Specifically, Crissolo, Ostana, Sanfront, and Pellice upstream sampling sites had a perennial flow regime compared with Revello, Pellice downstream, and Varaita which frequently experienced prolonged drying events.

**Table 1:** Occurrence of drying events in the intermittent sampling sites selected for the experiment. Pellice River did not experience extended dry events during the experiment, but only very low flow; however, previously collected data reported that dry events occurred in this river at the downstream site (Pellice V)

Stream	Sampling site	Time	Date	Hydrology
Po River	Revello	T0	13 Dec 2018	Flow
		T1	03 Jan 2019	Flow
		T2	24 Jan 2019	Low flow
		T3	14 Feb 2019	Dry
		T4	07 Mar 2019	Dry
		T5	29 Mar 2019	Dry
		T6	19 Apr 2019	Low flow
Pellice River	Pellice V	T0	13 Dec 2018	Flow
		T1	03 Jan 2019	Flow
		T2	24 Jan 2019	Low flow
		T3	14 Feb 2019	Low flow
		T4	07 Mar 2019	Flow
		T5	29 Mar 2019	Flow
		T6	19 Apr 2019	Flow
Varaita River	Varaita	T0	13 Dec 2018	Flow
		T1	03 Jan 2019	Flow
		T2	24 Jan 2019	Dry
		T3	14 Feb 2019	Low flow
		T4	07 Mar 2019	Low flow
		T5	29 Mar 2019	Low flow
		T6	19 Apr 2019	Low flow

To measure leaf litter decomposition associated microbial communities, leaf litter bags were employed using two leaf species abundant in the watersheds and with distinct characteristics of quality/recalcitrance: chestnut (*Castanea sativa*) with 47.64 ± 0.47 C:N content at the beginning of the experiment, and oak (*Quercus robur*), 38.98 ± 0.47 initial C:N content, both collected in autumn after abscission. For more details regarding the experimental setting, including additional data on leaf litter decomposition rates and associated macroinvertebrates, please see previous publications by Gruppuso et al. [42, 43]. Three leaf bags per leaf species were collected on each sampling date, placed into individual plastic bags, and stored at −20°C until DNA extraction and microbial community sequencing.

Frozen leaf litter samples were thawed and sampled using a cork-borer under sterile conditions. For each sample, 1 g of leaf tissue was stored in cryovials with Sigma-Aldrich RNAlater^®^ and kept frozen at −20°C. DNA extractions were performed using Qiagen MagAttract^®^ DNA Extraction Kit, following the manufacturer’s instructions except for the addition of lysozyme (15 mg/ml) during the lysis step. For bacterial communities, the V4 hypervariable region of the 16S rRNA gene [44–46] was amplified using dual-indexed Illumina compatible primers 515f/806r as described by Kozich JJ [47]. For fungal communities, we used ITS4 + ITS7 primers complementary to the internal transcribed spacer (ITS) region [48–50]. PCR products were batch-normalized using Invitrogen SequalPrep DNA Normalization plates and the products recovered from the plates were pooled. The pool was cleaned using an Amicon Spin Filter and AmpureXP magnetic beads. Samples were quantified with a combination of Qubit dsDNA HS, Agilent 4200 TapeStation HS DNA 1000, and Kapa Illumina Library Quantification qPCR assays.

Library preparation and sequencing (2 × 250 bp reads) were performed at the Michigan State Research Technology Support Facility on the Illumina MiSeq platform. The sample pool was loaded onto an Illumina MiSeq v2 standard flow cell and sequencing was performed in a 2×250 bp paired-end format using a MiSeq v2 500 cycle reagent cartridge. Custom sequencing and index primers complementary to the amplicon sequences were added to appropriate wells of the reagent cartridge as described in [47]. Base calling was done by Illumina Real Time Analysis (RTA) v1.18.54 and the output of RTA was demultiplexed and converted to FastQ format with Illumina Bcl2fastq v2.20.0.a.

### Data Processing and Statistical Analysis

Raw sequence files were demultiplexed and quality-filtered using default settings in DADA2 and QIIME 2 (2020.11; see [51, 52]). Taxonomy was assigned to amplicon sequencing variants (ASVs) using a Naive Bayes classifier. For bacterial amplicons, the SILVA (13.8; see [53]) reference dataset was used for classification while the UNITE (v8.99) database was used for fungal reads [54]. Shannon diversity was calculated using the phyloseq package [55] and compared using ANOVA tests or paired Wilcoxon tests depending on the comparison. Differences in taxonomic relative composition among groups were compared with Kruskal-Wallis and Wilcoxon tests and a false discovery rate (FDR) correction for multiple comparisons.

Differences in beta diversity were compared with PERMANOVA conducted using the vegan package [56], Bray-Curtis dissimilarity, and 999 permutations. Random forest models were built using genus-level taxonomy and out of bag (OOB) error to predict leaf and river type using the random forest package (v 4.6-14; see [57]). Only bacterial and fungal genera comprising more than 1% of total relative abundance across all samples were included in random forest modeling. Figures were created using a combination of ggplot2 (3.3.5), phyloseq (1.36.0), ggpubr (v 0.4.0), and vegan (v 2.5-7) packages (see [55, 58, 59]. All analyses were conducted using R (v 4.1.3; [60]). R code used in this study has been deposited on GitHub (https://github.com/JPReceveur/AlpineStreamMicroGruppuso2022). Sequencing data has been deposited in the NCBI SRA under the ascension number PRJNA882013.

## Results

### Bacterial Communities

Sequencing of 84 bacterial samples resulted in 6,262,055 reads with a mean reads per sample of 74,548 and 2993 unique ASVs. Based on alpha rarefaction curves, bacterial communities were rarefied to a depth of 7000 reads per sample.

### Impact of Flow Intermittency and Leaf Type on Leaf-Associated Microbial Communities

To examine the impact of flow intermittency on leaf bag microbial communities, four perennial sites (CRI, OST, PEM, and SAN) were compared with sites that frequently experience intermittent drying events (PEV, RE, and VAR).

Bacterial community Shannon diversity differed significantly by river type (ANOVA, perennial or intermittent, *P* < 0.001) and leaf type (chestnut or oak, *P* = 0.003) with a nearly significant effect of date (*P* = 0.098, Table S1). Both chestnut and oak leaf bags had significantly higher bacterial diversity in intermittent streams (*P* > 0.05, Table S2), with higher mean diversity for all dates except oak leaves at day 126 (Fig. S1a). While diversity in chestnut leaf bags was influenced by date (ANOVA, *F* =2.9, *P* = 0.029), increasing at later sampling days, oak bacterial diversity remained relatively constant over the study duration (*F*= 0.24, *P* = 0.94). When paired chestnut and oak leaf communities were compared (i.e., comparing leaf bags sampled at the same site and date), oak leaves had on average 0.24 (SEM +/− 0.04) higher values for Shannon diversity (Wilcoxon, *P* = 0.001, Fig. S1b) and 28.8 more ASVs (ASVs, +/− 11.0 SEM, *P* = 0.083, Chestnut = 407.0 +/− 16.2, Oak mean = 435.9 +/− 13.8).

**Fig. 1:**
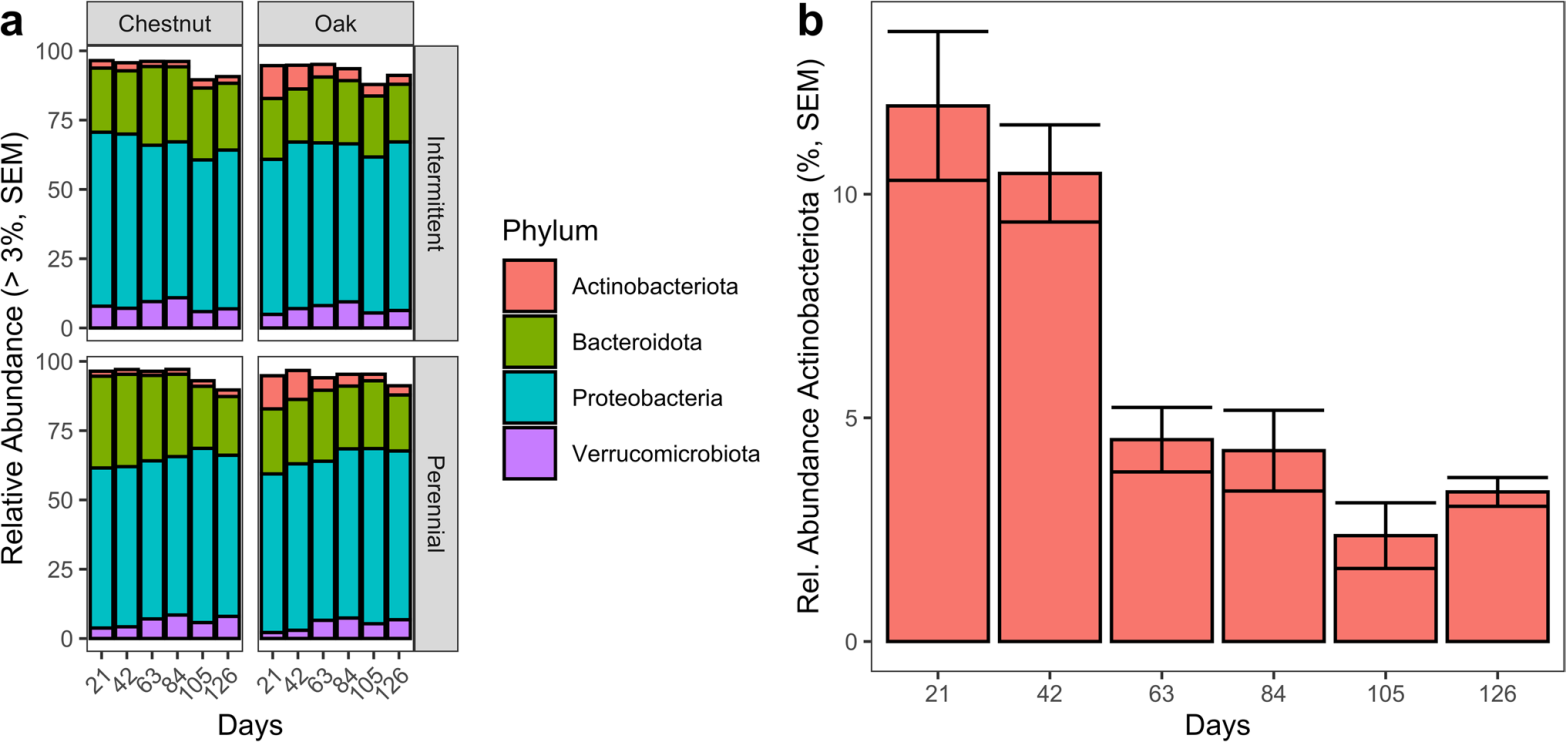
Leaf bag bacterial communities. **a** Relative bacterial abundance among samples from different leaf and river types. Only taxa which comprised greater than 3% of the total relative abundance are shown. **b** Relative abundance of Actinobacteriota in oak leaf bags from perennial rivers

Bacterial abundance at the phylum level was relatively consistent across dates for both perennial and intermittent sites with the phylum Proteobacteria comprising more than 50% of the relative abundance across all sample groups (Fig. 1a). For chestnut leaves, there were no phyla that differed significantly in relative abundance across dates for either river type (perennial or intermittent, KW, *P*-adj > 0.05) or between intermittent and perennial rivers (KW, *P*-adj > 0.05). In oak leaf bags, the relative abundance of Actinobacteriota was different among dates in perennial rivers (KW, *P*-adj = 0.014), with mean abundances on the first two sampling days (days 21 and 42) twice that of later dates (Fig. 1b); however, no differences in relative abundance were observed between river types (KW, *P* > 0.05). Similarly, there were no differences among the flow types (i.e., normal flow, low flow, dry/no flow, KW, *P*-adj > 0.05, Table 1) for either oak or chestnut leaves.

**Fig. 2:**
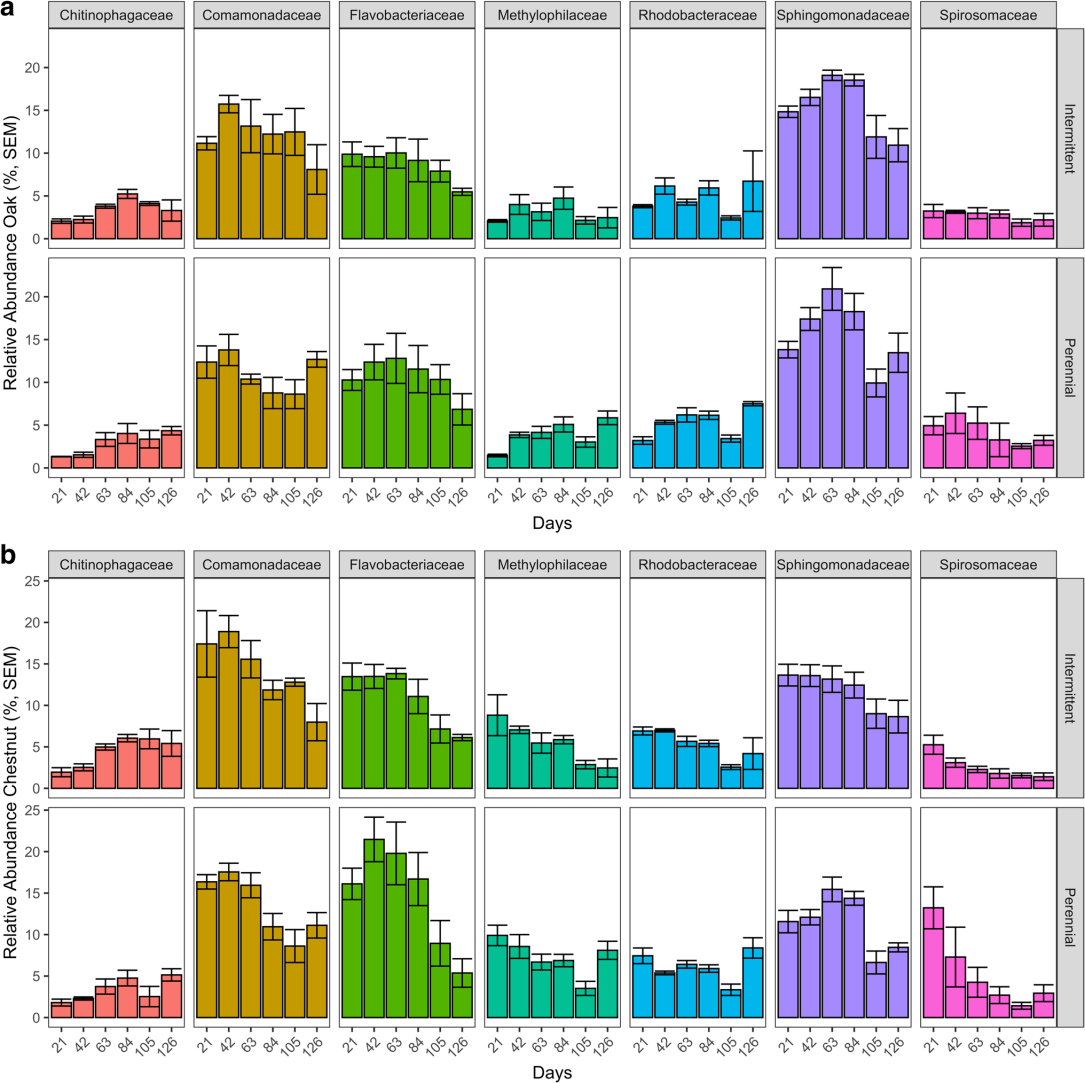
Family level bacterial abundance. **a** Oak bacterial abundance by date. **b** Chestnut bacterial abundance by date. Error bars are SEM. Only bacterial families that were greater than 3% of the total relative abundance across all samples and differed significantly for at least one comparison are shown (Kruskal-Wallis *p*-adj < 0.05). A list of comparisons is available in Table S3

**Fig. 3:**
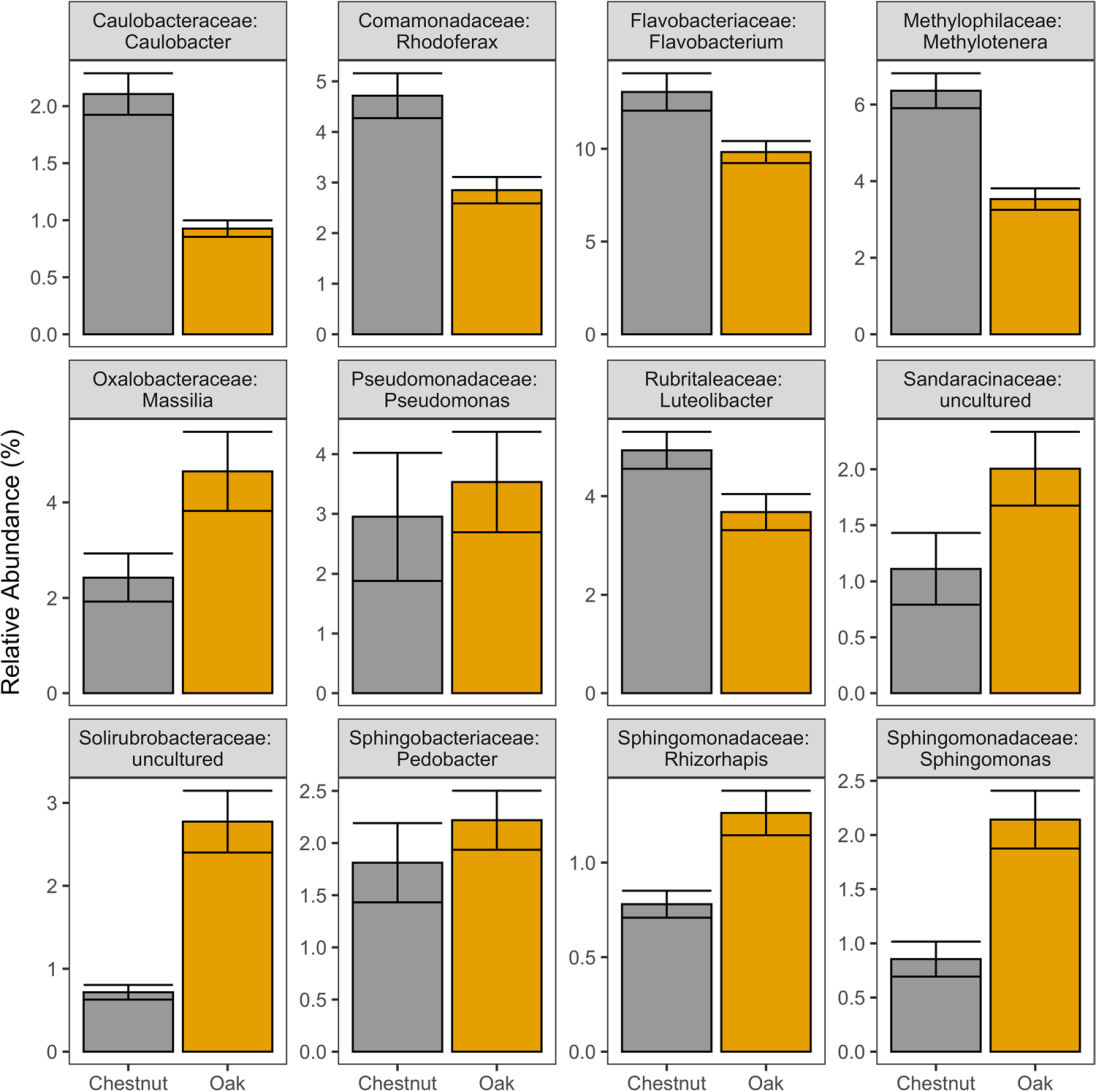
Top genus level random forest indicators for predicting leaf type. Error bars are SEM. Samples from different river types are combined

At the family level, bacterial communities were broadly similar, with few differences between river or leaf type (KW, *P*-adj > 0.05, Fig. 2a, b), though several taxa differed significantly in abundance across sampling dates (e.g., Sphingomonadaceae decreasing at dates 105 and 126, KW, *P*-adj < 0.05, Table S3). Bacterial genus-level community composition analyses revealed highly distinct communities between oak and chestnut leaf bags, with 94% (79/84) of samples correctly classified to leaf species (random forest, OOB error). All of the top 12 predictor genera (mean decrease accuracy score > 5, out of 21 genera comprising greater than 1% of total relative abundance) were significantly different between leaf types, with *Caulobacter* and *Methylotenera* higher in chestnut leaves, while *Massilia* and *Sphingomonas* representing oak leaf communities (Wilcoxon, *P*-adj > 0.05, Fig. 3, Table S4). Collectively, the top 12 predictor genera comprised 40.6% of the total relative community across all samples.

Within individual leaf litter species, the differences between bacterial communities of perennial and intermittent rivers were less distinct, with only 66% (28/42) of chestnut leaves and 64% of oak leaves (27/42) correctly classified to river type in random forest models. Of the top predictors within chestnut leaves, the abundance of only one group (an unassigned genus from the family Verrucomicrobiaceae) significantly differed between river types (KW, *P*-adj = 0.038, Fig. S2). Within oak leaves, only the genus *Caulobacter* (Family: Caulobacteraceae) was significantly different (KW, *P*-adj < 0.05) between intermittent (rel. abu. = 1.14% +/− 0.1) and perennial river leaf litter bacterial communities (rel. abu. = 0.77% +/− 0.1, Fig. S2).

### Bacterial Beta Diversity

**Fig. 4:**
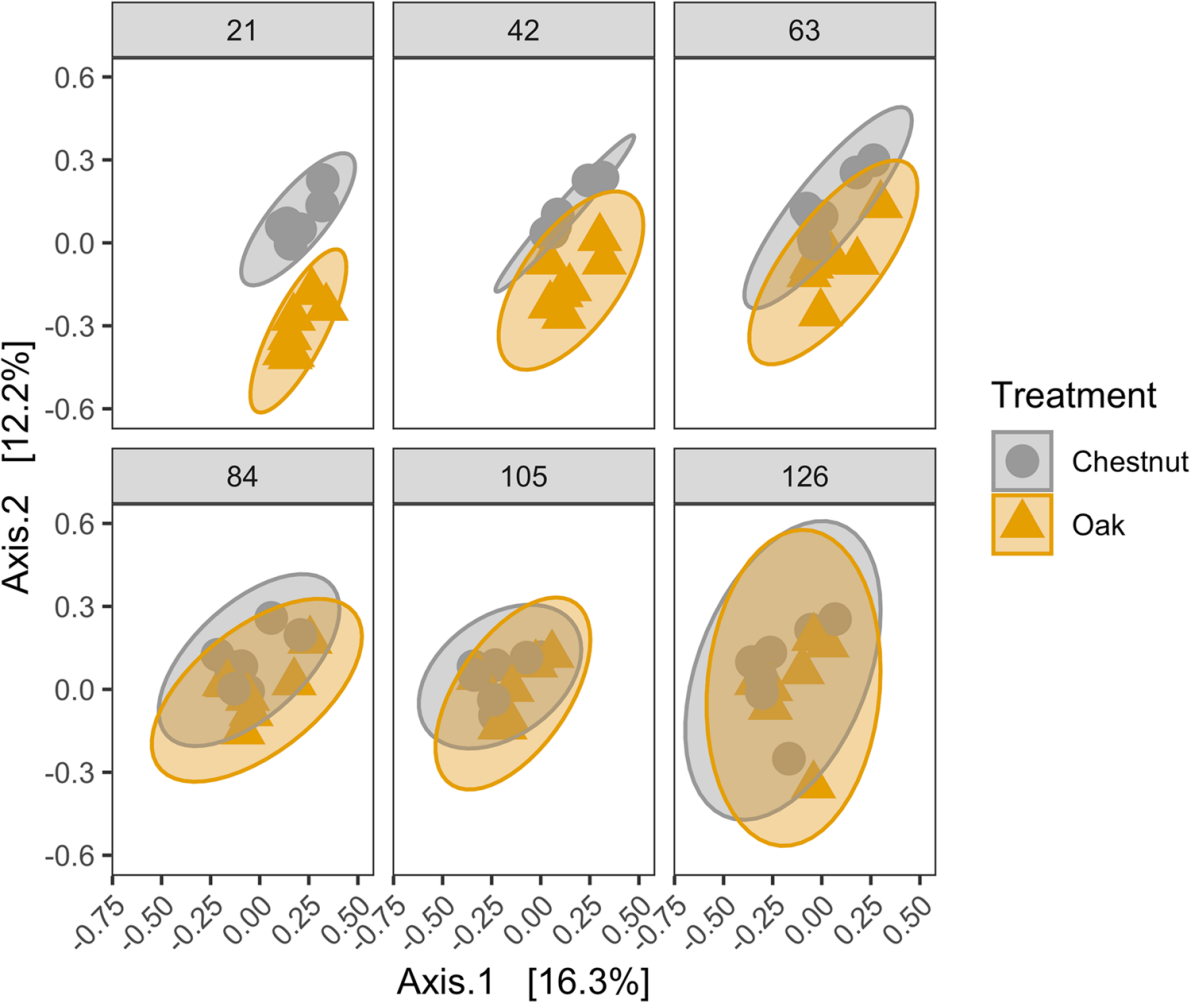
Difference in bacterial beta diversity between leaf types over time. PCoA is shown using Bray-Curtis Dissimilarity. Ellipses represent 95% confidence intervals for the mean of each group

Bacterial beta diversity (Bray-Curtis dissimilarity) was assessed by river/leaf types as well as site and date. River type, leaf type, site, and sampling day all had significant effects on bacterial community structure (PERMANOVA, *F* > 6.0, *P* < 0.001, Table S5) with leaf type (chestnut vs oak) having the largest effect size (*F*= 11.1, *P* < 0.001); however, the magnitude of difference between chestnut and oak communities changed over time (Fig. 4). At early sampling timepoints (days 21, 42, and 63), chestnut and oak communities clustered distinctly from each other and were significantly different when tested in a pairwise fashion (*P* < 0.05, Table S6). However, at later dates (days 84, 105, and 126), bacterial community structure was not significantly different between leaf types (*P* > 0.05). All sampling sites showed similar levels of variability in beta diversity (i.e., beta dispersion), with no site having significantly higher or lower variation than other sites (Vegan:Betadispr, *F*= 0.49, *P* = 0.82).

### Fungal Results

Across all fungal samples (*N* = 84), 1038 fungal ASVs were identified with over half (558, 54%) shared between communities of both leaf types, though some were unique: oak (257 unique ASVs, 25%), chestnut (223 unique ASVs, 21%). Fungal samples were rarefied to 6000 reads based on alpha diversity rarefaction curves. Time had a significant effect on fungal diversity with later dates (days 105 and 126) showing higher diversity for both oak and chestnut leaves than earlier dates (Fig. S3a, Table S7). River type did not strongly affect chestnut fungal community diversity (ANOVA, *F* = 1.98, *P* = 0.17), but oak leaf communities from intermittent rivers had higher diversity than leaf litter communities of perennial rivers (*F*= 4.47, *P* = 0.004). When oak and chestnut leaf communities were compared from the same site and date, oak leaves had higher Shannon diversity values (mean difference = 0.29 +/− 0.07 SEM) than chestnut leaves (Wilcoxon, *P* = 0.0003, Fig. S3b).

### Fungal Taxonomic Composition

**Fig. 5:**
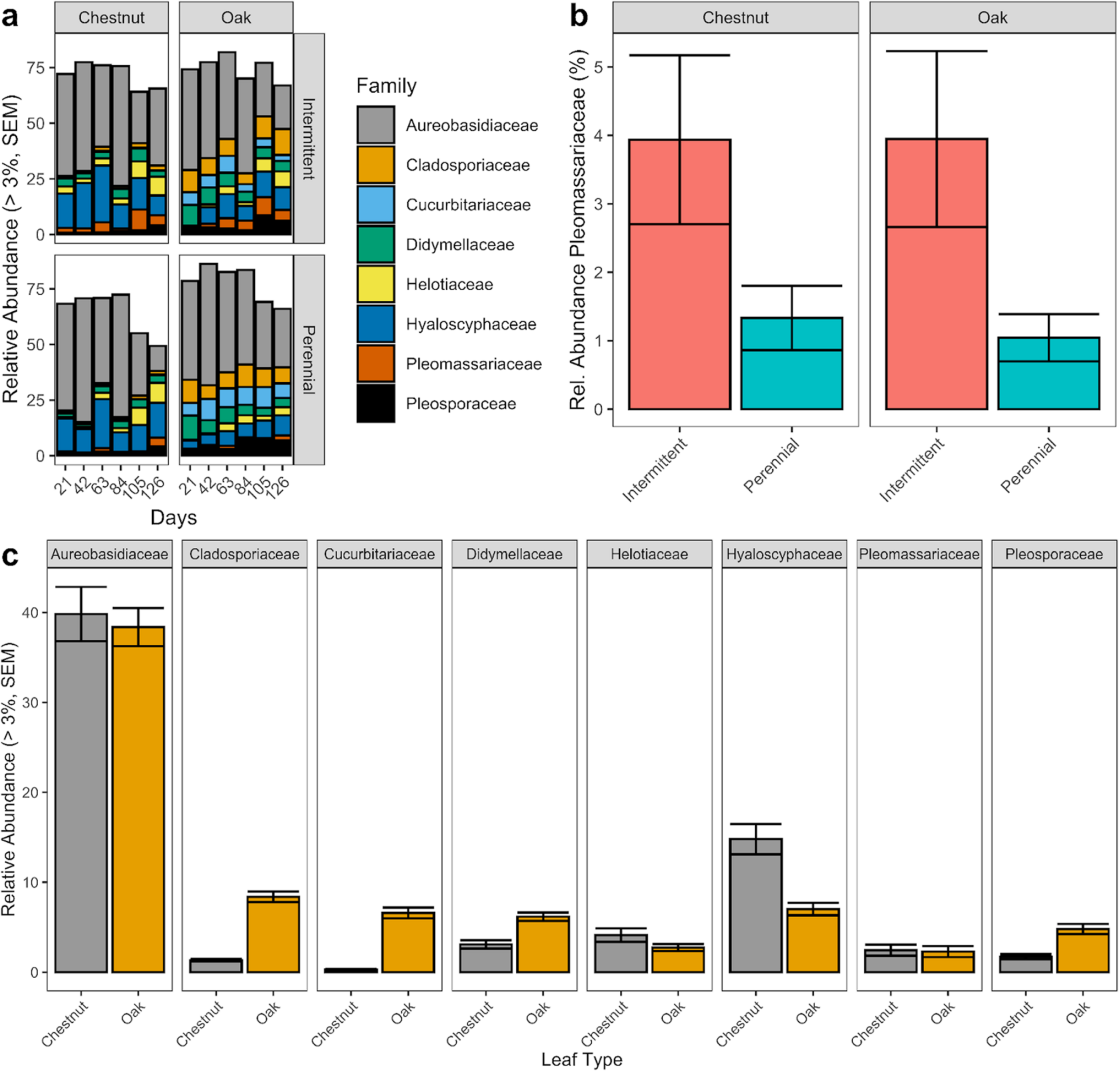
Family level fungal abundance. **a** Family level abundance across sampling day by leaf and river type. Only families which comprise greater than 3% of the relative abundance across all samples are shown. **b** Relative abundance of the family Pleomassariaceae (+/− SEM) between river types. **c** Differences in family level relative abundance between leaf types (both river types combined). Error bars are SEM

Ascomycota was the dominant fungal group across all samples representing 90.5% (SEM +/− 1.1) of the relative abundance across all samples. At the phylum level, none of the four most abundant phyla (Ascomycota, Basidiomycota, Chytridiomycota, and Mortierellomycota) differed in abundance between intermittent and perennial rivers for either chestnut or oak leaf communities (Fig. S4, KW, *P*-adj > 0.05). Communities described at a family level were similar between river types (Fig. 5a), with only one family (Pleomassariaceae) significantly different in relative abundance between perennial (1.33% +/− 0.47) and intermittent (3.94% +/− 1.23) rivers for chestnut leaves (Fig. 5b, KW, adj-*P* < 0.05). No fungal families differed significantly in abundance between rivers for oak leaf communities (*P*-adj > 0.05), though Pleomassariaceae (*P*-adj = 0.077) displayed a similar trend as chestnut leaves, with higher abundances in intermittent rivers (3.94 +/− 1.29 vs 1.04 +/− 0.34). There were several families which differed in abundance between chestnut and oak leaves including Cladosporiaceae, Cucurbitariaceae, Didymellaceae, Hyaloscyphaceae, and Pleosporaceae (Table S8, Fig. 5c, KW, *P*-adj > 0.001). Of those families, Hyaloscyphaceae had a higher relative abundance in chestnut leaf communities (14.8 +/−1.7 vs 7.0 +/− 0.69), while the remaining families were in higher abundance in oak leaves.

**Fig. 6:**
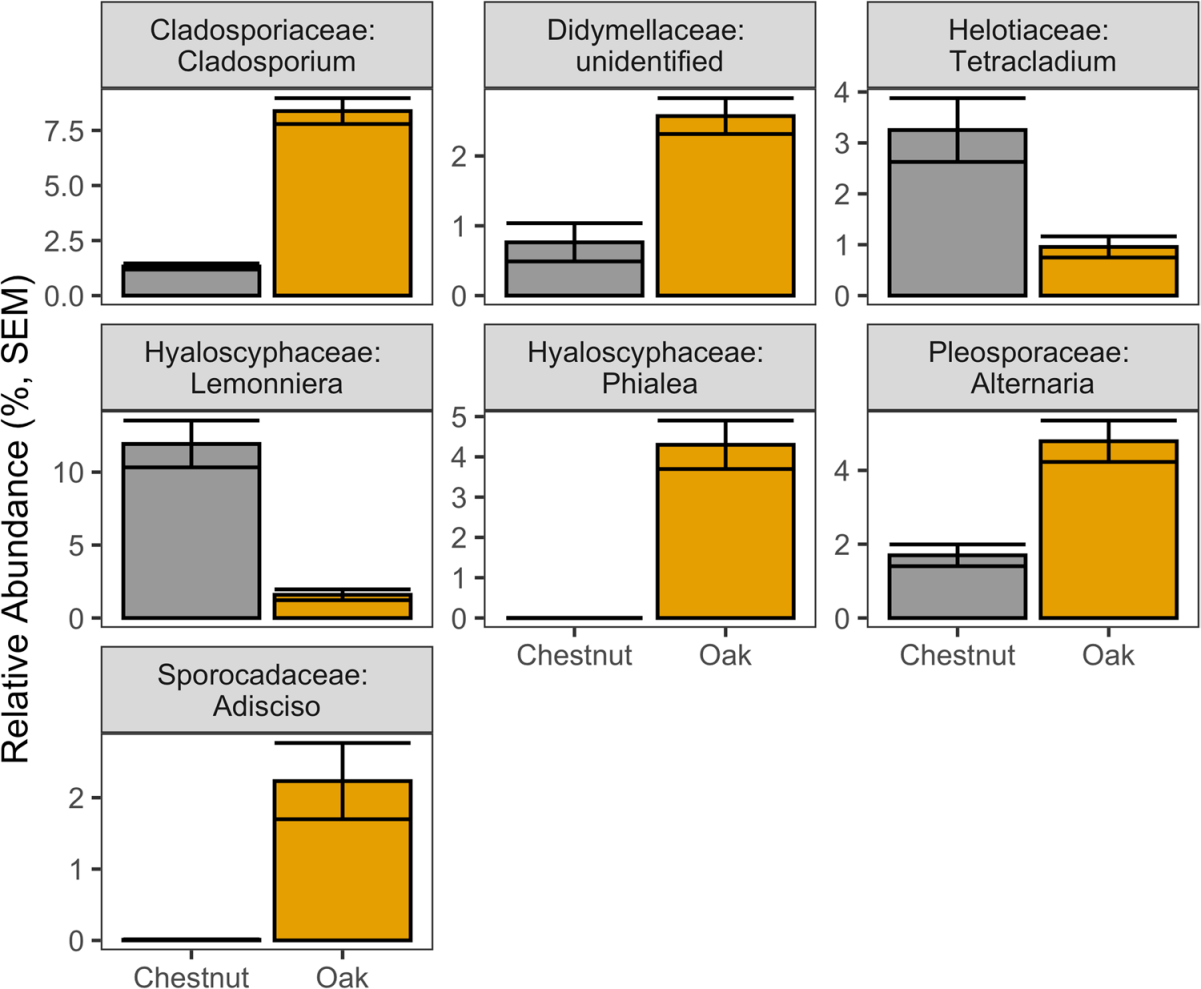
Top fungal genus level indicators for leaf type. Top indicators were identified by a Mean Decrease Accuracy (MDA) score greater than 5. All comparisons were significantly different (KW, *P*-adj < 0.01). Error bars are SEM

Genus-level fungal communities were highly distinct between leaf types with only a single sample misclassified to leaf type (random forest, OOB error = 1.19%). All of the top genus level indicator taxa (*N* = 7, of 13 fungal genera comprising greater than 1% of total relative abundance) in the random forest model (determined by a mean decrease accuracy score > 5) were significantly different between chestnut and oak leaf fungal communities (KW, *P*-adj < 0.01, Fig. 6, Table S9). The top fungal indicator taxa comprised 43.8% of the total relative abundance across all samples. Genus-level fungal communities were less distinct between river and flow conditions with models doing a poor job of classifying fungal communities to both river type (intermittent vs perennial, 30/84 samples misclassified) and flow condition (all dry samples and 87% of low flow samples misclassified).

### Fungal Beta Diversity

**Fig. 7:**
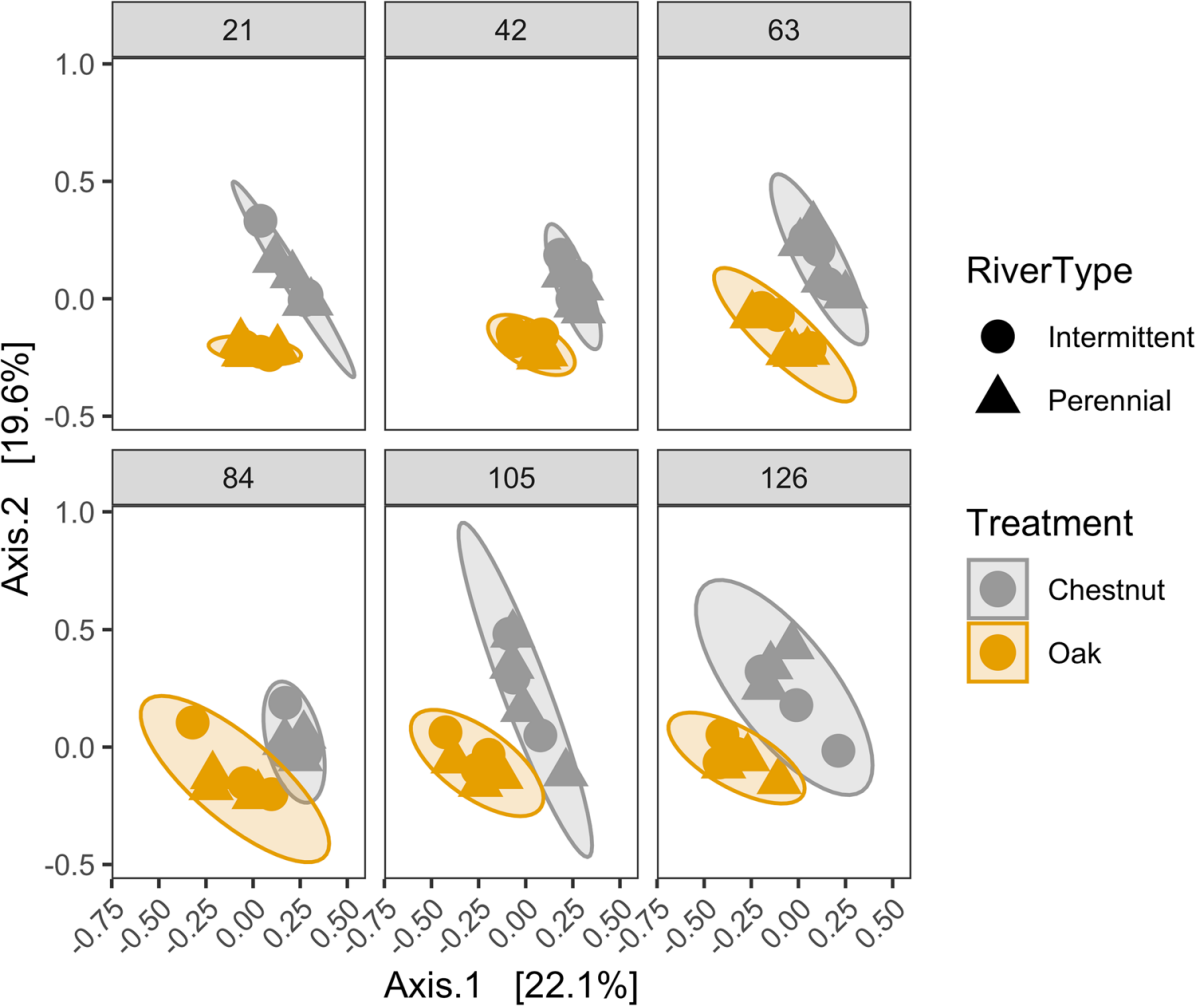
PCoA plot of fungal beta diversity between leaf type and river type (intermittent vs perennial). Ellipses represent 95% CI for the mean of each leaf species

Fungal community structure (Bray-Curtis dissimilarity) was significantly affected by flow conditions (dry, low flow, normal), leaf type, site, and date (PERMANOVA, *P* < 0.05, Table S10). Leaf type (chestnut vs oak) had the highest effect size (*F* = 25.03) and explained the greatest proportion of variation in beta diversity (*R*^2^ = 0.19) followed by the date (*F* = 4.47, *R*^2^ = 0.17, Fig. 7). River type (*F* = 2.48, *R*^2^ = 0.02, Table S10) and flow conditions (*F* = 2.86, *R*^2^ = 0.04, Fig. S5, Table S11) explained small proportions of beta diversity variation.

## Discussion

In this study, we investigated how bacterial and fungal community composition changed with flow regime for two common species of leaf litter. Our findings suggest that in Alpine stream conditions certain bacteria and fungi taxa are somewhat resilient to drying events. One explanation may be that fungal taxa originally present on the leaves (such as Cladosporaceae and Pleosporaceae [61–63]), or as early colonizers after submergence, may provide increased surface area and leachate that support specific microbial taxa growth and resistance to desiccation on either leaf type [64, 65]. The same may be the case for microbial taxa having differential responses to the production of trehalose by fungi that could have facilitated the resilience of certain taxa to drying events [66]. Differential taxon-specific responses to increased surface area, leachate quality, and compound production by early microbes may explain overall community diversity responses to drying. Certain taxa may remain resilient due to unknown trait differences (such as resistance spores and biofilm formation [67–69]) in these highly complex and changing assemblages during leaf decomposition.

Intermittent sites were characterized by higher abundances of microbial taxa with known desiccation resilience or the ability to use atmospheric oxygen to degrade aromatic compounds [70–72], such as Rhodobacteraceae, *Novosphingobium* and Pleomassariaceae [73, 74]. This group of fungi deserves additional investigation for a better understanding of how such taxa affect leaf litter decomposition processes during longer desiccation phases. Flow conditions affected fungal beta-diversity, but not to the extent that was expected. We found that the fungal community was mainly represented by Ascomycetes, and this may be because other groups, such as Basidiomycetes, often occur later in leaf litter decomposition, at stages we did not investigate during our field experiment [75]. The family Aureobasidiaceae, which is widespread in freshwater and marine ecosystems [76, 77], was the most abundant in our samples, regardless of leaf type and flow regime, likely because this family is known to adapt to different environmental conditions, and thus can cope with hydrological and nutrient stressors [76].

However, both bacterial and fungal β-diversity were significantly affected by flow conditions, suggesting that drying events and associated hydrological intermittence may play an important role in shaping the microbial community structure of decaying chestnut and oak leaves. Indeed, the intrinsic characteristics of the leaves and their species-specific microbial community at abscission [78, 79] are fundamental factors shaping initial community composition prior to entering the aquatic habitat, as previously reported for Mediterranean rivers [80]. For instance, oak (*Q. robur)* leaves were characterized by fungi commonly present in decomposing plant material (*Cladosporium*, *Alternaria*, *Phialea* and *Adisciso* [76, 81, 82]) and bacteria involved in aromatic compound degradation (Comamonadaceae, Actinobacteriota [83]) or able to use atmospheric oxygen during drying events (such as *Massilia*), possibly facilitating the decomposition of this leaf type (if active) even during flow intermittency. The higher bacterial and fungal diversity (increased evenness) [65, 84] of oak leaf communities suggests that recalcitrance and high secondary compound content may require a more complex microbial community for initial decomposition and mineralization [85]. In our previous work of leaf litter decomposition along the Po river [42, 43], we observed that chestnut leaves decomposed faster than oak, where the process continued, but delayed, during riverbed drying. Given the previous findings, combined with these microbial community changes, we suggest that terrestrial leaf-associated fungal taxa, along with early aquatic colonizers of in-stream litter accelerate decomposition for less recalcitrant leaves. Chestnut (*C. sativa)* leaves, however, were mainly characterized by species common in soil ecosystems or on the leaves themselves (*Caulobacter*, *Brevundimonas* and *Pedobacter*), thus not profoundly and negatively affected by desiccation [70, 72, 73] or nutrient scarcity [86]. Moreover, aquatic hyphomycetes and other decomposers (i.e., *Lemonniera* and *Tetracladium* [87, 88]), mainly involved in breakdown processes in aquatic environments, may explain the faster decomposition rate we found for chestnut leaves.

High-throughput sequencing of microbial communities associated with in-stream leaf litter reveals the complexity of microbial diversity, community composition, and species interactions on aquatic CPOM decomposition processes. In Alpine river ecosystems, the occurrence of low flow phases and drying events is a recent phenomenon, caused mainly by global climate change and anthropogenic pressures [89]. Previous studies confirm the hypothesis that the biological communities of these streams are not yet adapted to the phenomenon [37, 38, 90], and this may have negative effects on the allochthonous organic matter decomposition process, altering Alpine stream food webs. Microbial communities are fundamental to aquatic ecosystems, with their activity providing nutrients for the other organisms; however, our results showed that changing flow conditions did not strongly affect all bacterial and fungal taxa. Some families and genera may have been dormant (non-replicating and functioning) with detection only by the presence of their DNA. Therefore, simple detection does not necessarily mean that they are playing an active role in decomposition. Additional experimental studies on bacterial and fungal function during leaf litter decomposition are needed to address this possibility. Such studies could be performed with stream mesocosms to allow manipulations of the severity and length of drying events. Moreover, studying a complex phenomenon such as climate change at a broader level will require including cross-biome impacts, such as initial terrestrial leaf processing and subsequent changes within aquatic ecosystems. In stream systems, it will be important to understand the role of the terrestrial leaf microbial community at abscission and how that pioneer community drives subsequent community assembly of the leaf material in the aquatic habitat [15]. Better understanding the impact of riverbed drying on microbial communities is fundamental within the perspective of global climate change, especially in ecosystems where this phenomenon has not historically occurred.

## Supplementary Information

ESM 1 (DOCX 630 kb)
