# Supplementary material for: Hidden Decomposers: the Role of Bacteria and Fungi in Recently Intermittent Alpine Streams Heterotrophic Pathways
Source: Microb Ecol. 2023 Jan 17;86(3):1499–512. doi: 10.1007/s00248-023-02169-y (PMC10497695; doi:10.1007/s00248-023-02169-y)
Supplement: Supplementary file 1 — (DOCX 630 kb) [file 248_2023_2169_MOESM1_ESM.docx]

HIDDEN DECOMPOSERS: THE ROLE OF BACTERIA AND FUNGI IN RECENTLY INTERMITTENT ALPINE STREAMS HETEROTROPHIC PATHWAYS

Gruppuso L.^1,2*^, Receveur J. P.^3*^, Fenoglio S.^1,2^, Bona F.^1,2^, Benbow M. E.^4,5,6^

Corresponding author: Laura Gruppuso, [laura.gruppuso@unito.it](mailto:laura.gruppuso@unito.it)

**Supplementary material**

**Table S1** ANOVA comparison of bacterial Shannon diversity. Model = Shannon Diversity ~ River Type x Leaf Type x Days. Df = Degrees of Freedom, SS = Sum of Squares, MS = Mean Squares

|  | **Df** | **SS** | **MS** | **F** | **P** |
| --- | --- | --- | --- | --- | --- |
| River Type | 1 | 2.145 | 2.145 | 16.666 | <0.001 |
| Leaf Type | 1 | 1.26 | 1.26 | 9.788 | 0.003 |
| Days | 5 | 1.262 | 0.252 | 1.961 | 0.098 |
| RiverType:Leaf Type | 1 | 0.026 | 0.026 | 0.203 | 0.654 |
| RiverType:Days | 5 | 0.718 | 0.144 | 1.116 | 0.362 |
| LeafType:days | 5 | 0.872 | 0.174 | 1.355 | 0.254 |
| RiverType:LeafType:Days | 5 | 0.184 | 0.037 | 0.286 | 0.919 |
| Residuals | 60 | 7.722 | 0.1287 |  |  |

**Table S2** Bacterial Shannon diversity by river and date for each leaf type. ANOVA model = Bacterial diversity ~ River Type * Date. Results are shown separately for each leaf type

| **Chestnut** | **Model Term** | **Df** | **SS** | **MS** | **F** | **P** |
| --- | --- | --- | --- | --- | --- | --- |
|  | River Type | 1 | 1.322 | 1.3222 | 9.674 | 0.00408 |
|  | Days | 5 | 1.992 | 0.3984 | 2.915 | 0.02921 |
|  | River Type:Days | 5 | 0.491 | 0.0982 | 0.718 | 0.61469 |
|  | Residuals | 30 | 4.1 | 0.1367 |  |  |
| **Oak** | **Model Term** | **Df** | **SS** | **MS** | **F** | **P** |
|  | River Type | 1 | 0.849 | 0.8488 | 7.031 | 0.0127 |
|  | Days | 5 | 0.142 | 0.0284 | 0.235 | 0.9438 |
|  | River Type: Days | 5 | 0.411 | 0.0822 | 0.681 | 0.6411 |
|  | Residuals | 30 | 3.622 | 0.1207 |  |  |

**Table S3** Differences in family level bacterial relative abundance over time separated by leaf and river type. Kruskal-Wallis test with a FDR comparison was used to identify taxa that differed by day

| **Leaf Type** | **River Type** | **Family** | **p** | **p.adj** |
| --- | --- | --- | --- | --- |
|  |  | Pseudomonadaceae | 0.135 | 0.16 |
|  |  | **Flavobacteraceae** | 0.0201 | 0.044 |
|  |  | **Comamonadaceae** | 0.0117 | 0.044 |
|  |  | **Sphingomonadaceae** | 0.00577 | 0.044 |
|  |  | **Spirosomaceae** | 0.023 | 0.044 |
|  | Perennial | Oxalobacteraceae | 0.148 | 0.16 |
|  |  | **Methylophilaceae** | 0.0276 | 0.044 |
|  |  | **Rhodobacteraceae** | 0.0282 | 0.044 |
|  |  | Rubritalaceae | 0.207 | 0.21 |
|  |  | Chitinophagaceae | 0.0401 | 0.055 |
| Chestnut |  | Caulobacteraceae | 0.0184 | 0.044 |
|  |  | Oxalobacteraceae | 0.805 | 0.8 |
|  |  | Comamonadaceae | 0.057 | 0.094 |
|  |  | Flavobacteriaceae | 0.0601 | 0.094 |
|  |  | Sphingomonadaceae | 0.205 | 0.25 |
|  |  | Methylophilaceae | 0.0274 | 0.094 |
|  | Intermittent | Caulobacteraceae | 0.0372 | 0.094 |
|  |  | Rubritaleaceae | 0.157 | 0.22 |
|  |  | Pseudomonadaceae | 0.391 | 0.43 |
|  |  | Chitinophagaceae | 0.057 | 0.094 |
|  |  | **Rhodobacteraceae** | 0.0451 | 0.094 |
|  |  | Spirosomaceae | 0.0521 | 0.094 |
|  |  | Pseudomonadaceae | 0.0618 | 0.11 |
|  |  | **Sphingomonadaceae** | 0.0217 | 0.08 |
|  |  | Flavobacteriaceae | 0.447 | 0.45 |
|  |  | Comamonadaceae | 0.13 | 0.2 |
|  |  | **Oxalobacteraceae** | 0.0355 | 0.098 |
|  |  | Spirosomaceae | 0.399 | 0.44 |
|  |  | **Rhodobacteraceae** | 0.00228 | 0.025 |
|  | Perennial | **Methylophilaceae** | 0.00915 | 0.05 |
|  |  | Rubritaleaceae | 0.198 | 0.24 |
|  |  | Chitinophagaceae | 0.0608 | 0.11 |
|  |  | Caulobacteraceae | 0.143 | 0.2 |
|  |  | Oxalobacteraceae | 0.279 | 0.56 |
|  |  | **Sphingomonadaceae** | 0.016 | 0.18 |
|  |  | Comamonadaceae | 0.41 | 0.56 |
|  |  | Flavobacteriaceae | 0.178 | 0.49 |
| Oak |  | Rhodobacteraceae | 0.138 | 0.49 |
|  |  | Oxalobacteraceae | 0.279 | 0.56 |
|  |  | **Sphingomonadaceae** | 0.016 | 0.18 |
|  |  | Comamonadaceae | 0.41 | 0.56 |
|  |  | Flavobacteriaceae | 0.178 | 0.49 |
|  |  | Rhodobacteraceae | 0.138 | 0.49 |
|  | Intermittent | Caulobacteraceae | 0.756 | 0.76 |
|  |  | Rubritaleaceae | 0.537 | 0.59 |
|  |  | Pseudomonadaceae | 0.367 | 0.56 |
|  |  | Methylophilaceae | 0.485 | 0.59 |
|  |  | **Chitinophagaceae** | 0.0476 | 0.26 |
|  |  | Spirosomaceae | 0.377 | 0.56 |

**Table S4** Top genus level indicators for leaf type. Top indicators were chosen using a Mean Decrease Accuracy Score > 5. p.adj indicates a FDR corrected p value. Kruskal-Wallis test was used for comparison

| **Family: Genus** | **p** | **p.adj** |
| --- | --- | --- |
| Caulobacteraceae: Caulobacter | 2.22E-07 | 2.70E-06 |
| Flavobacteriaceae: Flavobacterium | 0.01256199 | 0.013 |
| Rubritaleaceae: Luteolibacter | 0.0122487 | 0.013 |
| Oxalobacteraceae: Massilia | 0.00039612 | 0.00079 |
| Methylophilaceae: Methylotenera | 3.15E-06 | 1.30E-05 |
| Sphingobacteriaceae: Pedobacter | 0.00525177 | 0.0063 |
| Pseudomonadaceae: Pseudomonas | 0.00112848 | 0.0017 |
| Spingomonadaceae: Rhizorhapis | 0.00196589 | 0.0026 |
| Comamonadaceae: Rhodoferax | 0.00112848 | 0.0017 |
| Sphingomonadaceae: Sphingomonas | 2.53E-06 | 1.30E-05 |
| Sandaracinaceae: uncultured | 0.00016657 | 4.00E-04 |
| Solirubrobacteraceae: uncultured | 1.32E-05 | 4.00E-05 |

**Table S5** Difference in bacterial beta diversity between groups. DF = degrees of freedom, SS = Sum of Squares, MS = Mean Squares. Model = Distance matrix ~ River Type x Leaf Type x Site + Date. PERMANOVA test used 999 permutations

|  | **DF** | **SS** | **MS** | **F** | **R2** | **P** |
| --- | --- | --- | --- | --- | --- | --- |
| River Type | 1 | 1.76 | 1.18 | 10.15 | 0.07 | <0.001 |
| Leaf Type | 1 | 1.29 | 1.29 | 11.1 | 0.07 | <0.001 |
| Site | 5 | 0.76 | 0.76 | 6.53 | 0.21 | <0.001 |
| Date | 5 | 0.72 | 0.72 | 6.26 | 0.2 | <0.001 |
| River Type: Leaf Type | 1 | 0.08 | 0.08 | 0.73 | 0.004 | 0.78 |
| Leaf Type: Site | 5 | 0.38 | 0.08 | 0.66 | 0.02 | 0.99 |
| Residuals | 65 | 7.53 | 0.11 |  |  |  |

**Table S6** Comparison of bacterial beta diversity between leaf types within individual dates

| **Day** | **Model Term** | **DF** | **SS** | **MS** | **F** | **R2** | **P** |
| --- | --- | --- | --- | --- | --- | --- | --- |
| 21 | Leaf Type | 1 | 0.91 | 0.91 | 7.23 | 0.38 | 0.002 |
|  | Residuals | 12 | 1.5 | 0.13 |  |  |  |
| 42 | Leaf Type | 1 | 0.42 | 0.42 | 3.1 | 0.21 | <0.001 |
|  | Residuals | 12 | 1.62 | 0.13 |  |  |  |
| 63 | Leaf Type | 1 | 0.29 | 0.29 | 1.95 | 0.14 | 0.04 |
|  | Residuals | 12 | 1.78 | 0.15 |  |  |  |
| 84 | Leaf Type | 1 | 0.27 | 0.27 | 1.68 | 0.12 | 0.083 |
|  | Residuals | 12 | 1.91 | 0.16 |  |  |  |
| 105 | Leaf Type | 1 | 0.17 | 0.17 | 0.81 | 0.06 | 0.65 |
|  | Residuals | 12 | 2.53 | 0.21 |  |  |  |
| 126 | Leaf Type | 1 | 0.18 | 0.18 | 0.81 | 0.06 | 0.6 |
|  | Residuals | 12 | 2.67 | 0.22 |  |  |  |

**Table S7** Fungal Shannon diversity by river type (perennial vs intermittent) and sampling date. ANOVA model = Fungal diversity ~ River Type * Date. Results are shown separately for each leaf type

|  | **Model Term** | **Df** | **SS** | **MS** | **F** | **P** |
| --- | --- | --- | --- | --- | --- | --- |
|  | River Type | 1 | 0.348 | 0.348 | 1.98 | 0.17 |
|  | Days | 5 | 3.94 | 0.788 | 4.47 | 0.004 |
| **Chestnut** | River Type:  Days | 5 | 0.461 | 0.09 | 0.52 | 0.76 |
|  | Residuals | 30 | 5.29 | 0.18 |  |  |
|  | River Type | 1 | 0.522 | 0.522 | 4.66 | 0.038 |
|  | Days | 5 | 2.1 | 0.42 | 3.75 | 0.009 |
| **Oak** | River Type:  Days | 5 | 0.197 | 0.0395 | 0.353 | 0.877 |
|  | Residuals | 30 | 3.358 | 0.111 |  |  |

**Table S8** Differences in family level fungal communities between leaf bag type (Oak vs Chestnut). Samples were combined across day and river types. P-adj indicates a Kruskal-Wallis test with a FDR correction for multiple comparisons

| **Family** | **P-adj** | **Leaf Type** | **Mean** | **SEM** |
| --- | --- | --- | --- | --- |
| Aureobasidiaceae | 0.57 | Chestnut  Oak | 39.8  38.4 | 3.02  2.12 |
| Cladosporiaceae | < 0.001 | Chestnut  Oak | 1.3  8.4 | 0.14  0.59 |
| Cucurbitariaceae | < 0.001 | Chestnut  Oak | 0.3  6.6 | 0.06  0.62 |
| Didymellaceae | < 0.001 | Chestnut  Oak | 3.1  6.2 | 0.47  0.46 |
| Helotiaceae | 0.52 | Chestnut  Oak | 4.1  2.7 | 0.75  0.39 |
| Hyaloscyphaceae | < 0.001 | Chestnut  Oak | 14.8  7.0 | 1.79  0.69 |
| Pleomassariaceae | 0.18 | Chestnut  Oak | 2.4  2.3 | 0.62  0.62 |
| Pleosporaceae | < 0.001 | Chestnut  Oak | 1.7  4.8 | 0.30  056 |

**Table S9** Top fungal genus level indicators for leaf type. Top random forest indicators were chosen using a Mean Decrease Accuracy Score > 5. Adj-P value indicates a FDR corrected p value. Kruskal-Wallis test was used for comparison

| **Family: Genus** | **Treatment** | **mean** | **se** | **P** | **Adj-P value** |
| --- | --- | --- | --- | --- | --- |
| Cladosporiaceae: Cladosporium | Chestnut | 1.32 | 0.14 | 4.96E-15 | 3.50E-14 |
|  | Oak | 8.38 | 0.59 |  |  |
| Didymellaceae: unidentified | Chestnut | 0.76 | 0.27 | 7.95967E-10 | 1.1E-09 |
|  | Oak | 2.57 | 0.25 |  |  |
| Helotiaceae: Tetracladium | Chestnut | 3.25 | 0.62 | 0.000621528 | 0.00062 |
|  | Oak | 0.95 | 0.21 |  |  |
| Hyaloscyphaceae: Lemonniera | Chestnut | 11.93 | 1.60 | 1.33566E-10 | 2.3E-10 |
|  | Oak | 1.59 | 0.37 |  |  |
| Hyaloscyphaceae: Phialea | Chestnut | 0 | 0 | 6.46E-13 | 2.30E-12 |
|  | Oak | 4.29 | 0.60 |  |  |
| Pleosporaceae: Alternaria | Chestnut | 1.69 | 0.29 | 1.55E-09 | 1.80E-09 |
|  | Oak | 4.79 | 0.56 |  |  |
| Sporocadaceae: Adisciso | Chestnut | 0.0096 | 0.0051 | 1.70E-12 | 4.00E-12 |
|  | Oak | 2.23 | 0.53 |  |  |

**Table S10** PERMANOVA test comparing fungal beta diversity (Bray-Curtis dissimilarity) between groups. PERMANOVA model: Distance matrix ~ RiverType*Leaf Type*Site+Date. DF= Degrees of Freedom. SS = Sum of Squares. MS = Mean Squares

|  | **DF** | **SS** | **MS** | **F** | **R2** | **P** |
| --- | --- | --- | --- | --- | --- | --- |
| River Type | 1 | 0.2719 | 0.27186 | 2.477 | 0.01806 | 0.016 |
| Leaf Type | 1 | 2.7946 | 2.79456 | 25.4621 | 0.18564 | 0.001 |
| Site | 5 | 1.5187 | 0.30375 | 2.7676 | 0.10089 | 0.001 |
| Date | 5 | 2.5244 | 0.50488 | 4.6002 | 0.16769 | 0.001 |
| River Type:  Leaf Type | 1 | 0.1165 | 0.11649 | 1.0614 | 0.00774 | 0.334 |
| Leaf Type:  Site | 5 | 0.6939 | 0.13877 | 1.2644 | 0.04609 | 0.099 |
| Residuals | 65 | 7.134 | 0.10975 |  | 0.4739 |  |
| Total | 83 | 15.0539 |  |  | 1 |  |

**Table S11** PERMANOVA test comparing fungal beta diversity (Bray-Curtis dissimilarity) between groups. PERMANOVA model: Distance matrix ~ RiverType*Leaf Type*Site+Date. DF= Degrees of Freedom. SS = Sum of Squares. MS = Mean Squares

|  | **DF** | **SS** | **MS** | **F** | **R2** | **P** |
| --- | --- | --- | --- | --- | --- | --- |
| River Type | 2 | 0.6387 | 0.31936 | 2.8599 | 0.04243 | 0.003 |
| Leaf Type | 1 | 2.7946 | 2.79456 | 25.0256 | 0.18564 | 0.001 |
| Site | 6 | 1.3829 | 0.23048 | 2.064 | 0.09186 | 0.001 |
| Date | 5 | 2.4963 | 0.49926 | 4.4709 | 0.16582 | 0.001 |
| River Type:Leaf Type | 2 | 0.2397 | 0.11985 | 1.0733 | 0.01592 | 0.352 |
| River Type:Site | 3 | 0.2955 | 0.09851 | 0.8822 | 0.01963 | 0.62 |
| Leaf Type:Site | 6 | 0.7813 | 0.13022 | 1.1662 | 0.0519 | 0.181 |
| Sample Type:Leaf Type:Site | 3 | 0.2831 | 0.09438 | 0.8452 | 0.01881 | 0.676 |
| Residuals | 55 | 6.1417 | 0.11167 |  | 0.40798 |  |
| Total | 83 | 15.0539 |  |  | 1 |  |

**Figure S1** Differences in bacterial Shannon diversity between intermittent and perennial leaf bags. **a)** Differences in Shannon diversity across days for each river type, split by leaf species. Error bars are Standard Error of the Mean (SEM). Panel **b)** shows paired comparisons between chestnut and oak leaf bags (i.e. between leaf bags sampled at the same site and date)


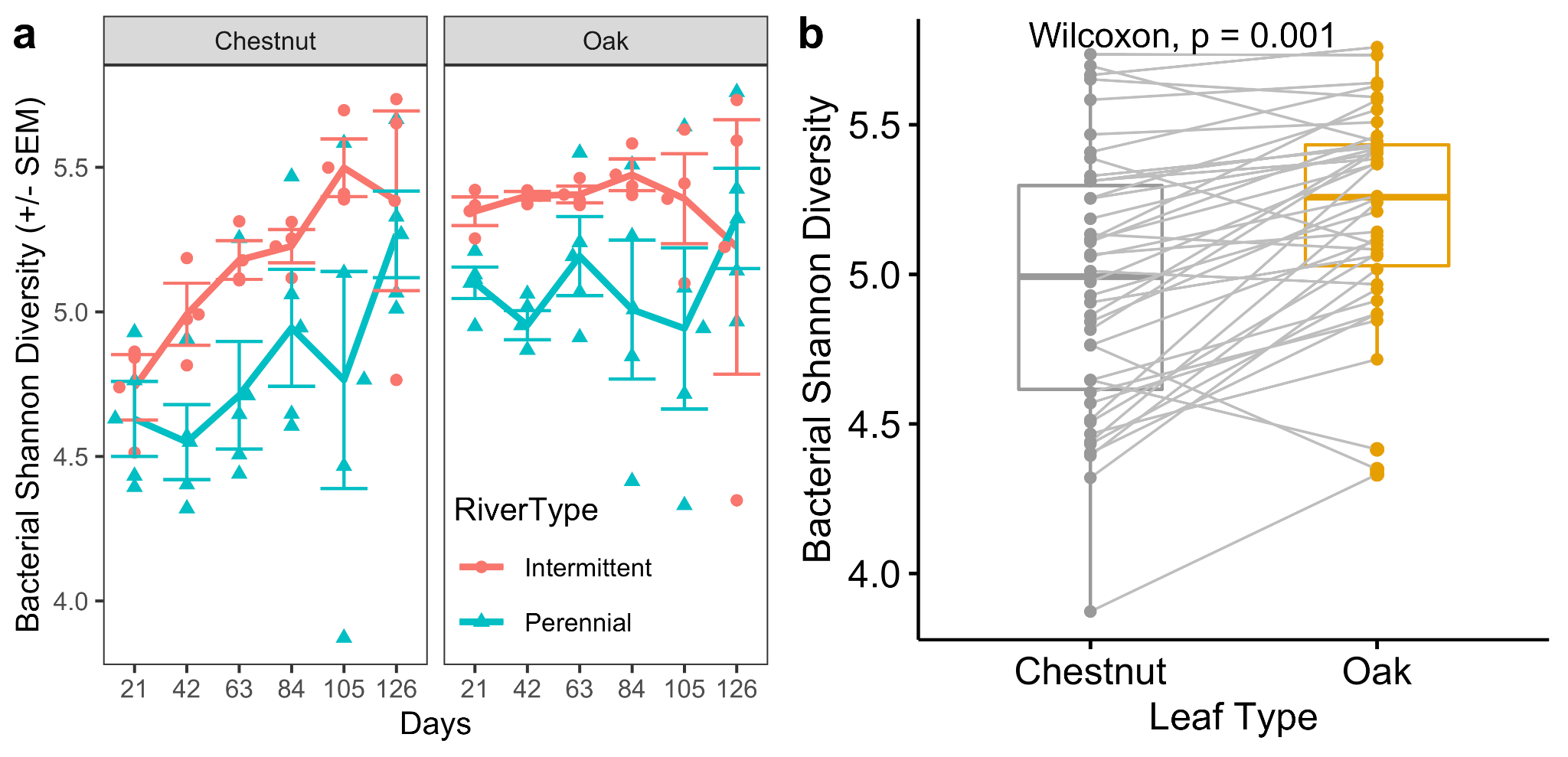


**Figure S2** Top bacterial genus level random forest indicators for predicting river type. **a)** Top predictors of river type for chestnut leaves. **b)** Top predictors of river type for oak leaves. Error bars are SEM


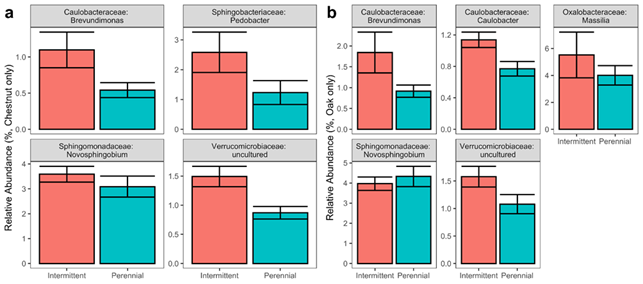


**Figure S3** Fungal Shannon diversity. **a)** Fungal Shannon diversity by leaf and river type across sampling days. Error bars are SEM. **b)** Difference in diversity between paired samples (i.e. between chestnut and oak samples taken from the same site and date). Lines connect paired samples


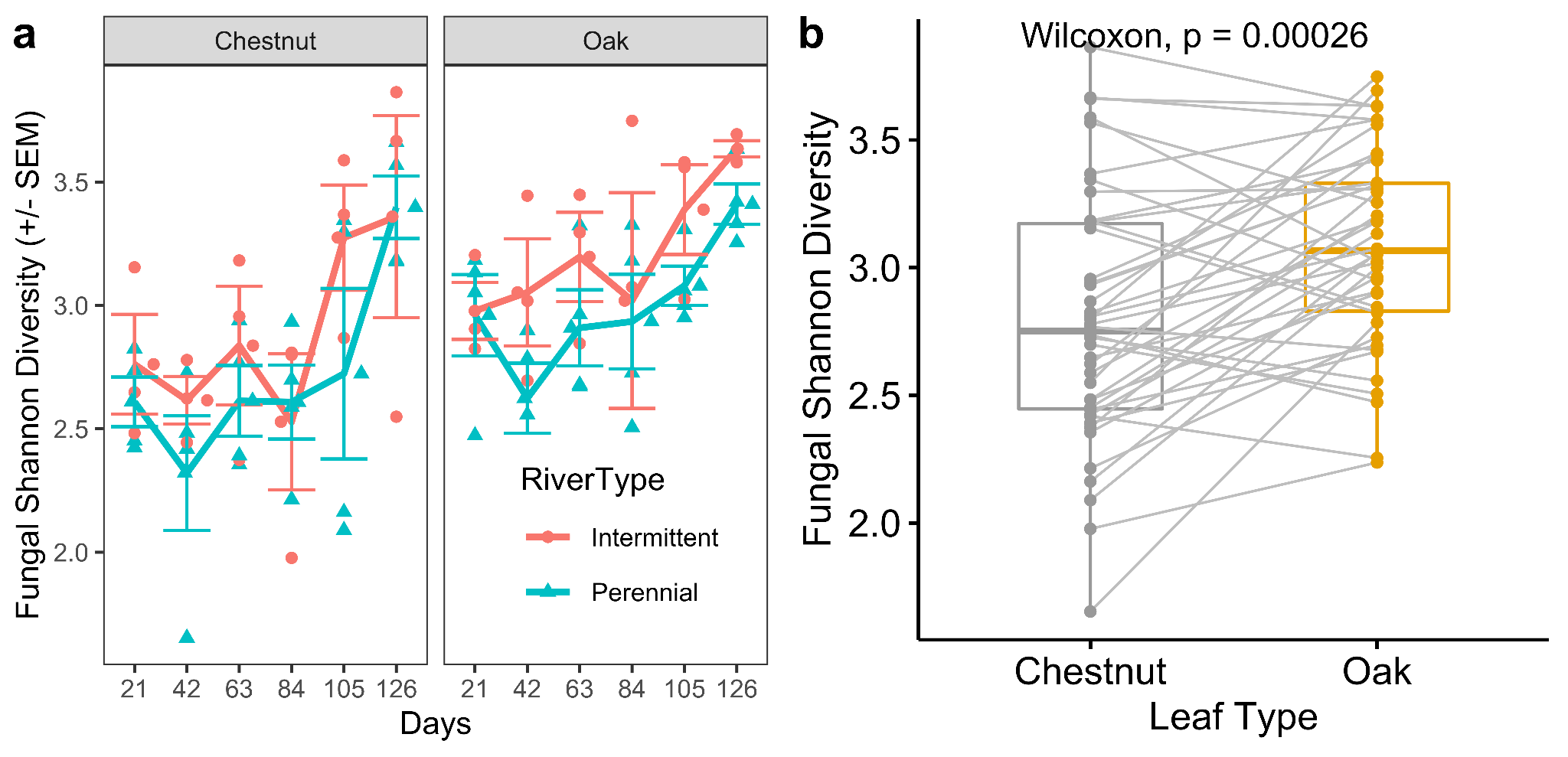


**Figure S4** Relative abundance of fungal phyla across sampling day and river type for each leaf species. Only phyla which comprised greater than 3% of the total relative abundance are shown


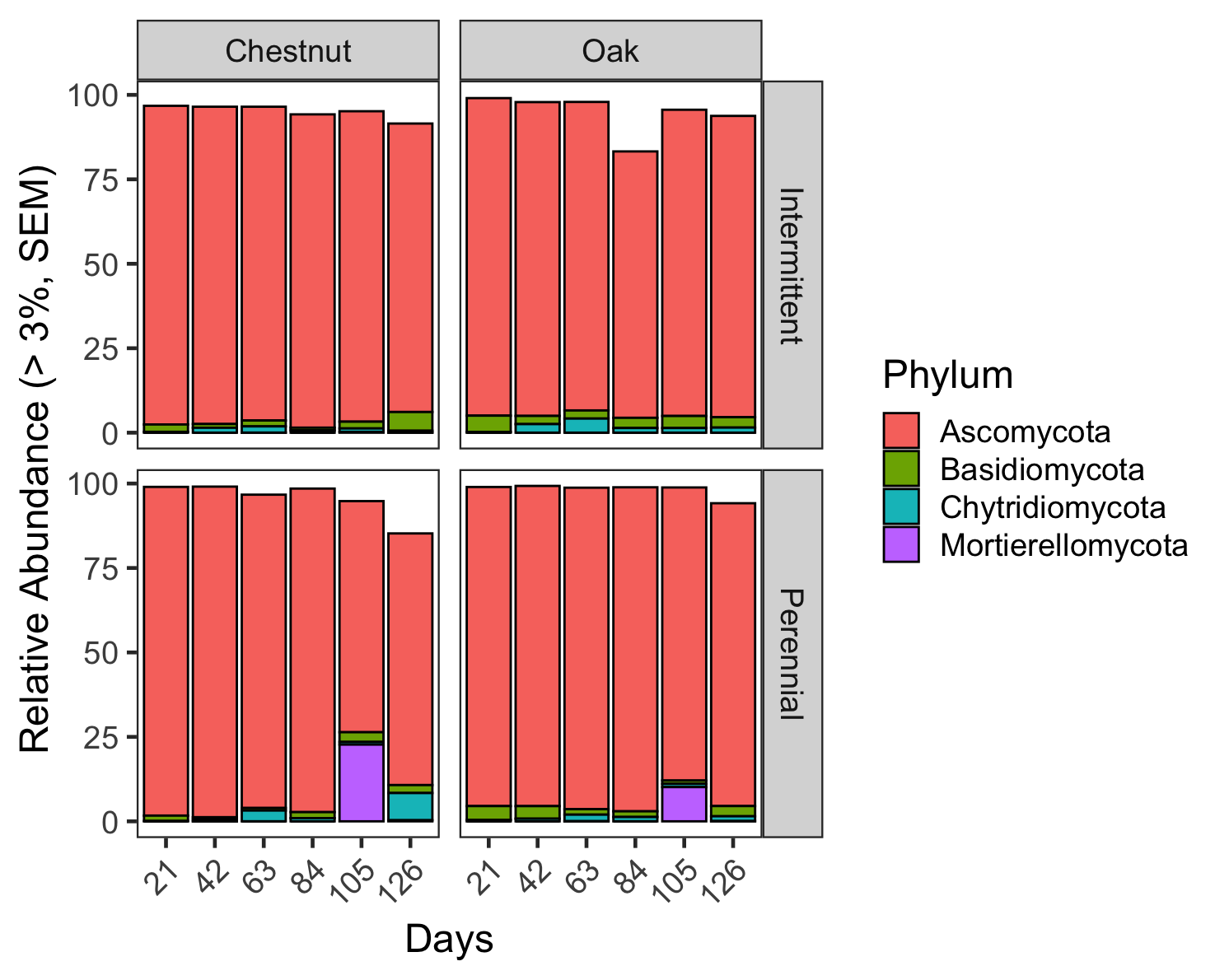


**Figure S5** PCoA plot of fungal beta diversity between leaf type and flow conditions (dry, low flow, normal flow). Ellipses represent 95% CI for the mean of each leaf species

*
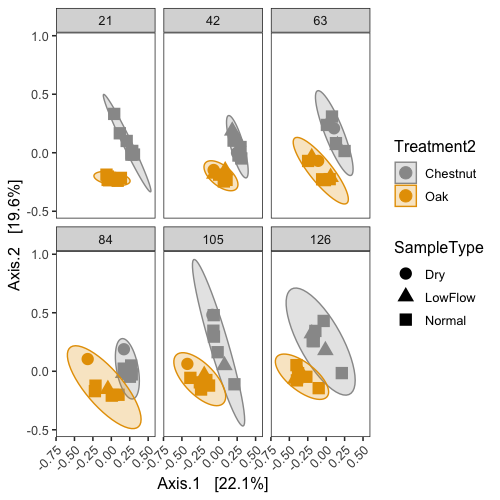
*
